# Human milk microbiota, oligosaccharide profiles, and infant gut microbiome in preterm infants diagnosed with necrotizing enterocolitis

**DOI:** 10.1016/j.xcrm.2024.101708

**Published:** 2024-08-30

**Authors:** Andrea C. Masi, Lauren C. Beck, John D. Perry, Claire L. Granger, Alice Hiorns, Gregory R. Young, Lars Bode, Nicholas D. Embleton, Janet E. Berrington, Christopher J. Stewart

**Affiliations:** 1 Translational and Clinical Research Institute, Newcastle University, Newcastle upon Tyne NE2 4HH, UK; 2 Microbiology Department, Freeman Hospital, Newcastle upon Tyne NE7 7DN, UK; 3 Newcastle Neonatal Service, Newcastle Hospitals NHS Trust, Newcastle upon Tyne NE1 4LP, UK; 4 Department of Pediatrics, Larsson-Rosenquist Foundation Mother-Milk-Infant Center of Research Excellence (MOMI CORE), University of California San Diego, La Jolla, CA 92093, USA; 5 The Human Milk Institute (HMI), University of California San Diego, La Jolla, CA 92093, USA; 6 Population Health Sciences Institute, Newcastle University, Newcastle upon Tyne NE2 4HH, UK

**Keywords:** milk microbiome, infant microbiome, gut microbiome, microbiota, preterm infant, necrotizing enterocolitis, multi-omics

## Abstract

Necrotizing enterocolitis (NEC) is a severe intestinal disease of very preterm infants with mother’s own milk (MOM) providing protection, but the contribution of the MOM microbiota to NEC risk has not been explored. Here, we analyze MOM of 110 preterm infants (48 NEC, 62 control) in a cross-sectional study. Breast milk contains viable bacteria, but there is no significant difference in MOM microbiota between NEC and controls. Integrative analysis between MOM microbiota, human milk oligosaccharides (HMOs), and the infant gut microbiota shows positive correlations only between *Acinetobacter* in the infant gut and *Acinetobacter* and *Staphylococcus* in MOM. This study suggests that NEC protection from MOM is not modulated through the MOM microbiota. Thus, “‘restoring” the MOM microbiota in donor human milk is unlikely to reduce NEC, and emphasis should instead focus on increasing fresh maternal human milk intake and researching different therapies for NEC prevention.

## Highlights

• Mother’s own milk microbiome and bacterial load are comparable between NEC and control
• No correlation exists between milk microbiome, HMO profile, and preterm gut microbiome
• *Acinetobacter* abundance positively correlates in milk microbiome and preterm gut

## Graphical abstract

**Figure:**
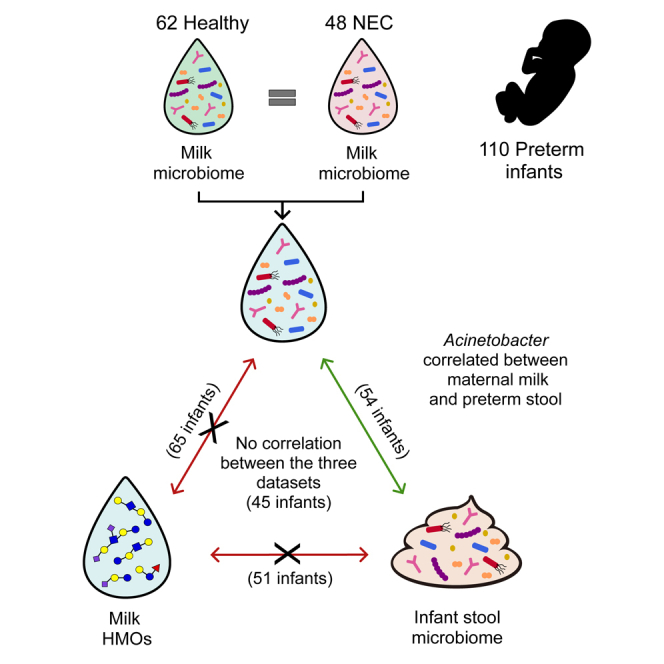

## Teaser

Masi et al. show that mother’s own milk bacterial composition and load are not altered in preterm infants diagnosed with necrotizing enterocolitis. Milk microbiome, human milk oligosaccharide, and preterm gut microbiome are not strongly correlated, suggesting other important factors also impact development of the preterm gut microbiome.

## Introduction

Necrotizing enterocolitis (NEC) is an inflammatory-mediated intestinal disease which is the leading cause of neonatal mortality in preterm infants born <32 weeks of gestation, affecting 5%–10% of this population.^1^ Despite decades of research, the mechanisms leading to NEC onset are unclear, but the preterm gut microbiota has been repeatedly associated with disease onset^2^^,^^3^ and new research suggests mother’s own milk (MOM) bioactive composition may also contribute.

MOM is a complex combination of nutrients, prebiotics, live microorganisms, and components with antimicrobial and immunomodulatory properties. Receipt of MOM is associated with a reduced NEC risk of up to 10-fold,^4^ yet infants exclusively fed MOM can still develop NEC,^5^ suggesting that differences in the composition of bioactive components between mothers might be responsible for variation in protective effects. In particular, lack of specific human milk oligosaccharides (HMOs) in MOM has been associated with NEC onset.^6^ HMOs are complex sugars abundant in human milk that cannot be digested by the infant, exerting their beneficial effects primarily by acting as a prebiotic.^7^ While there are more than 150 different structures described to date, work has focused on the most abundant, where one specific HMO, disialyllacto-N-tetraose (DSLNT), has been associated with a lower incidence of NEC. DSLNT was first found to reduce NEC-like disease in a neonatal rat model^8^ and subsequently in cohort studies of preterm infants from different geographical locations including South Africa,^9^ North America,^6^ and the UK.^10^

The preterm infant gut microbiome has been linked with NEC development, but no consistent microbial taxa have been associated with NEC onset.^1^ Overall, higher relative abundance of Pseudomonadota phylum and Enterobacteriales family has been linked to NEC,^10^^,^^11^^,^^12^ whereas a gut rich in *Bifidobacterium* spp. has been associated with health.^3^ Lower DSLNT concentration in MOM has been linked to a reduced transition of the preterm gut microbiota to communities dominated by bifidobacteria.^10^

In term and preterm infants, MOM microbiota has been reported as a potential source of bacteria that can colonize the infant gut.^13^^,^^14^^,^^15^ HMOs further shape the neonatal gut microbiota by acting as prebiotics, favoring *Bifidobacterium* spp. colonization.^16^ Moreover, HMOs might also modulate the MOM microbial community and vice versa, but the relationship between these two bioactive components *within* human milk requires further study.^17^^,^^18^^,^^19^

To date, studies focused on the role of MOM microbiota in NEC are lacking. Stewart et al. (2013) investigated the relationship between the microbiota of preterm twins and MOM microbiota, including twin pairs discordant for NEC development, but no direct investigation of MOM microbiota correlation with NEC was performed.^20^ Additionally, the potential relationship between MOM microbiota, HMO profiles, and preterm infant gut microbiota has not been investigated. Despite this, recent work has provided proof of concept that the MOM microbiota can “seed” pasteurized donor human milk,^21^^,^^22^^,^^23^ but whether this approach is likely to have any resulting benefit to health depends on whether MOM microbiota is associated with disease, which has not yet been explored.

In this study we aimed to investigate MOM microbiota in a large cohort of preterm (<32 weeks gestation at birth) NEC (*n* = 54 in total, 48 after rarefaction) and gestationally matched control infants (*n* = 72 in total, 62 after rarefaction). We hypothesized that a difference in MOM microbial composition and subsequent infant seeding was present between NEC and control infants. The MOM microbiota was profiled using amplicon-based 16S rRNA gene sequencing, and total bacterial load was determined with quantitative PCR (qPCR) for all samples that passed rarefaction. In a subset for whom MOM HMO profile (*n* = 65) and infant gut metagenome (*n* = 54) were available, an integration of the three datasets was performed.

## Results

### Overview of MOM microbiota composition

MOM samples from 54 NEC infants and 72 healthy controls underwent 16S rRNA gene sequencing. Samples were rarefied at 1,231 reads, retaining 62 control and 48 NEC samples for analysis. For the samples which passed rarefaction, the number of mapped reads of MOM had a median value of 30,251 (interquartile range [IQR] 4,660; 51,428) before rarefaction was applied. Seven kit negative controls were also sequenced, obtaining between 1 and 17 reads, and so all negatives were lost during rarefaction. Of the rarefied, retained samples, no significant differences in demographics were found between NEC and control groups across all co-variates (Table 1).

**Table 1:** Demographics of the mother’s own milk analytical cohort with cross-sectional 16S rRNA gene sequencing data that passed quality control

	Control	NEC	*p* value
Number of patients	62	48	–
Male	30 (48%)	31 (65%)	0.090
Vaginal delivery	36 (58%)	26 (54%)	0.683
Gestational age	26 [24; 27]	25 [24; 27]	0.703
Birth weight	800 [640; 900]	713 [589; 855]	0.129
DOL breast milk sample	19 [14.25; 28.75]	20 [14; 27]	0.758
DOL NEC onset	–	17.5 [12; 29.5]	–
MOM collected before NEC onset	–	25 (52%)	–
Developed LOS	15 (24%)	17 (35%)	0.199
LOS pre-NEC diagnosis	–	7 (15%)	–
LOS during NEC	–	2 (4%)	–
NEC surgical	–	23 (48%)	–
HMO profile available	36 (58%)	29 (60%)	–
Probiotics ever	62 (100%)	45 (94%)	0.204

Values are reported as median and inter-quartile ranges in brackets, or n (%). Differences between groups were tested applying chi-squared test and Wilcoxon Rank test where applicable. DOL, day of life; HMO, human milk oligosaccharide; LOS, late onset sepsis; NEC, necrotizing enterocolitis.

**Figure 1:**
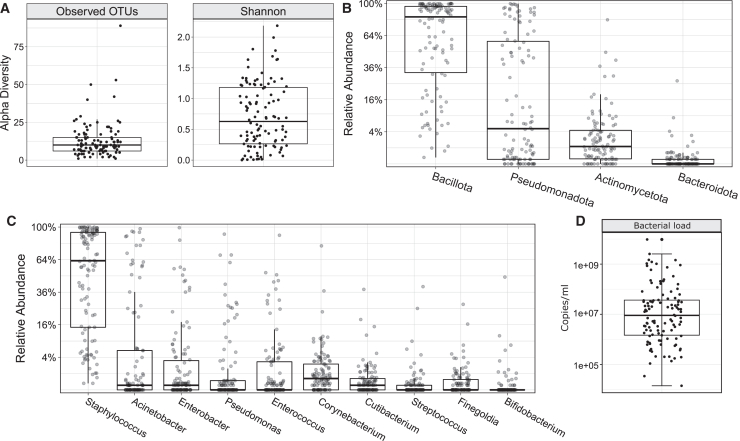
Cross-sectional overview of the MOM microbiota in preterm infants (A) Boxplots showing the alpha diversity based on observed OTUs (operational taxonomic unit; richness) and Shannon diversity. Boxplots showing the relative abundance of phyla (B) and the top 10 most abundant genera (C); square-root scaling was applied to the y axis. (D) Boxplot of the total bacterial load; log10 scaling was applied to the y axis. A total 110 preterm infants were included.

We first investigated the overall composition of the preterm MOM microbiota without stratifying by disease status. The median number of operational taxonomic units (OTUs) was 10 (IQR 6–15) with a median Shannon diversity of 0.63 (IQR 0.27–1.18) (Figure 1A). At the phylum level, Bacillota dominated (mean relative abundance 65.4%), followed by Pseudomonadota and Actinomycetota (mean 29% and 5.1%, respectively) (Figure 1B). At the genus level the 10 most abundant bacteria in rank order were *Staphylococcus* (57.1%), *Acinetobacter* (13.9%), *Enterobacter* (7%), *Pseudomonas* (5.7%), *Enterococcus* (5.4%), *Corynebacterium* (2.8%), *Cutibacterium* (1.2%), *Finegoldia* (0.9%), *Streptococcus* (0.9%), and *Bifidobacterium* (0.8%) (Figure 1C). *Bifidobacterium* was uncommon: 17/110 (15%) samples had relative abundance above zero, of which 13 had an abundance > 1%. The presence and viability of the top 10 most abundant genera were confirmed using traditional microbiological culture as described in the later sections.

We further characterized the MOM bacterial load by qPCR. The median bacterial load was 9.025e+06 copies/mL (IQR 1.467e+06; 3.669e+07), and a significant positive correlation was found between bacterial load and the number of 16S rRNA gene sequencing reads (R = 0.81, *p* < 0.001).

### All abundant bacteria in MOM could be isolated in culture

Microbiological culturing of MOM was used to confirm if the top 10 genera composing the MOM microbiota were viable. 16 MOM samples abundant in at least one of the top 10 genera identified through 16S rRNA gene sequencing were cultured, and up to 13 bacterial species were isolated in a single sample (Table S1). The most common species was *Staphylococcus epidermidis* (11/16, 69%), followed by *Cutibacterium acnes* (8/16, 50%) and *Enterococcus faecalis* (6/16, 38%). *Bifidobacterium breve*, *Enterobacter hormaechei*, *Staphylococcus lugdunensis*, and *Stenotrophomonas maltophilia* were in 3/16 samples (19%) (Table S1). Other *Bifidobacterium* spp. isolated included *B. animalis* (2/16, 13%), *B. longum* (1/16, 6%), and *B. bifidum* (1/16, 6%). *Staphylococcus* was the most abundant genus in MOM samples, and culturing supplemented the 16S rRNA gene sequencing results (limited to genus level) by revealing that multiple *Staphylococcus* spp. can coexist in the same sample, which was also observed for *Bifidobacterium*, *Acinetobacter*, and *Enterobacter* species.

### MOM microbiota does not correlate with NEC development

**Figure 2:**
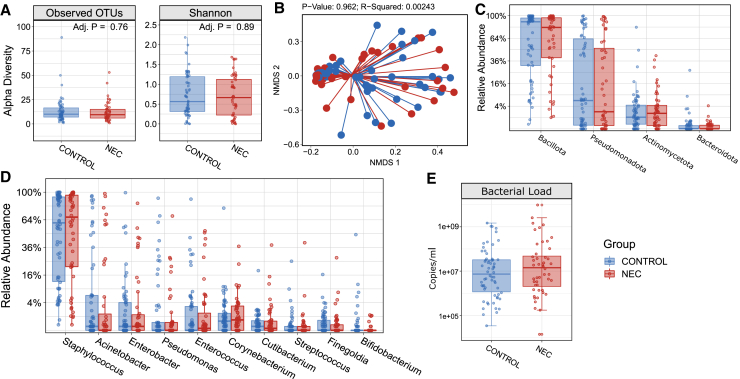
Analysis of mother’s own milk microbiota from preterm infants who were diagnosed with NEC and matched controls (A) Boxplots showing the alpha diversity based on observed OTUs (richness) and Shannon diversity. *p* values were calculated by applying the Mann-Whitney test and adjusted using the false discovery rate (FDR) algorithm. (B–D) (B) NMDS plot of weighted Bray-Curtis dissimilarity. *p* value based on permutational analysis of variance (PERMANOVA). Boxplots showing the relative abundance of phyla (C) and the top 10 most abundant genera (D); square-root scaling was applied to the y axis. (E) Boxplot of the total bacterial load; log10 scaling was applied to the y axis; *p* = 0.33. *p* values were calculated by applying the Mann-Whitney test and adjusted using the FDR algorithm. Adjusted *p* values in (C) and (D) were all >0.05. A total of 62 control and 48 NEC infants were included. See also Figures S1–S3.

We next sought to determine if MOM microbiota was associated with NEC development by comparing MOM received by infants who developed NEC (*n* = 48), with day of life (DOL)-matched controls (*n* = 62). Alpha diversity richness (median NEC 9.5 [IQR 6–15] vs. control 10 [IQR 7–16.5]; *p* = 0.7) and Shannon diversity (median NEC 0.67 [IQR 0.22–1.13] vs. control 0.56 [IQR 0.31–1.19]; *p* = 0.81) did not differ between MOM received by infants diagnosed with NEC compared to controls (Figure 2A). Beta diversity was also comparable (*p* = 0.937; Figure 2B). There was also no significant difference in the relative abundance of any phylum (all adj. *p* > 0.05; Figure 2C) or genus (all adj. *p* > 0.05; Figure 2D). Aside from the bacterial profiles, we also analyzed total bacterial load of all MOM samples that passed rarefaction and were included in the analytical dataset. Consistently, we found no significant difference in the bacterial load between NEC and control infants (*p* = 0.33, Figure 2E). To confirm this was not influenced by proximity of sampling in relation to disease, we next ran the analysis only on the subset of 25 infants with MOM samples collected before NEC diagnosis. Consistently, no difference was found in MOM alpha diversity, beta diversity, taxonomic relative abundance, or bacterial load between infants diagnosed with NEC and controls (Figure S1).

NEC can be divided into medically managed NEC (NEC-M) and surgically managed NEC (NEC-S), which relates to disease severity. Stratifying by NEC-M (*n* = 25) and NEC-S (*n* = 23) showed no statistically significant difference in MOM microbiota between NEC-S and NEC-M or when compared to matched no-NEC controls (Figure S2). Additionally, NEC infants MOM samples were also analyzed by whether NEC occurred before or after full enteral feeds (150 mL/kg/day sustained for 72 h) were achieved. This stratification of before (*n* = 29) and after (*n* = 18) accounts for differing age and maturity of the infant at the time of NEC diagnosis, since the potential gut microbial impact on disease is likely to differ. Consistently, no statistically significant associations were found for any analysis of MOM microbiota when stratifying NEC in relation to achieving full enteral feeds (Figure S3).

### HMO profiles, MOM microbiota, and infant gut microbiota are not strongly correlated

We were able to utilize previously generated HMO profiling data (*n* = 65)^10^ from a subset of exactly the same MOM samples used in the current study, as well as corresponding infant gut microbiome data from metagenomic sequencing (*n* = 54) (nearest available infant stool sample based on DOL MOM).^10^^,^^24^ Since no difference was found in MOM microbiota between NEC and healthy infants, subsequent analysis was performed independent of disease status to maximize power. Infant gut microbiota was analyzed at the genus level for direct comparison with MOM samples that underwent amplicon-based sequencing (i.e., limited to the genus level). In total, 65 MOM samples had both HMO and microbiota, 54 mother-infant pairs had MOM and infant stool microbiota data, 51 mother-infant pairs had MOM HMO profiles and infant stool microbiota data, and 45 pairs had one sample analyzed in each of the three datasets. The same MOM sample was used for both HMO and MOM microbiota, while the infant gut metagenome sample was usually also collected on the same day as the MOM sample (median number of days between the infant gut metagenome and MOM sample was 0 days; IQR 0–2).

**Figure 3:**
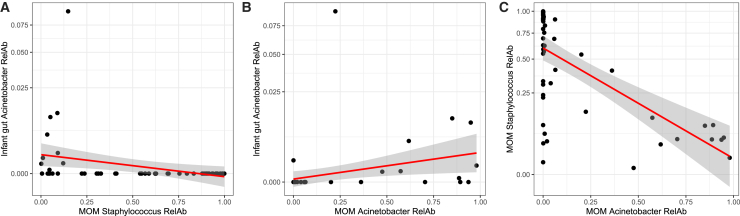
Correlation plots between the relative abundance of genera in the infant gut and mother’s own milk (A) Regression plot of MOM *Staphylococcus* relative abundance (RelAb) and infant *Acinetobacter* RelAb, *p* < 0.01. (B) MOM *Acinetobacter* RelAb and infant *Acinetobacter* RelAb, *p* < 0.01. (C) MOM *Acinetobacter* RelAb and MOM *Staphylococcus* RelAb, *p* < 0.001. For each panel, square-root scaling was applied to the y axis. R and *p* values were calculated by Spearman correlation analysis, and all *p* values were adjusted using the FDR algorithm. A total of 54 preterm infants were included.

The top 10 most abundant bacterial genera in MOM were tested for correlation with the same genera in matched infant stool (*Staphylococcus*, *Acinetobacter*, *Enterobacter*, *Pseudomonas*, *Enterococcus*, *Corynebacterium*, *Cutibacterium*, *Finegoldia*, *Streptococcus*, and *Bifidobacterium)*. A significant negative correlation was found between *Acinetobacter* in the infant gut and *Staphylococcus* in MOM (R = −0.52, adj. *p* < 0.01; Figure 3A), and a significant positive correlation was found between *Acinetobacter* in both samples (R = 0.50, adj. *p* < 0.01; Figure 2B). A significant negative correlation between *Acinetobacter* and *Staphylococcus* within MOM was also found (R = −0.57, adj. *p* < 0.001, Figure 3C). Notably, when outliers were removed from the analysis, correlations between infant gut *Acinetobacter* and MOM *Acinetobacter* and *Staphylococcus* relative abundances were not significant anymore. No other significant correlations were found.

Spearman correlation analysis was also performed between MOM microbiota and MOM HMO profiles, as well as infant gut microbiota and MOM HMO profiles. No significant correlation was found between any HMO and any of the top 10 genera in MOM or the infant gut microbiota (Figures S4A and S4B).

To further determine if there was any relationship between paired MOM and infant microbiotas, Bray-Curtis dissimilarity distances were calculated between mother-infant dyads compared to a randomly paired, unrelated, mother and infant samples. No differences in Bray-Curtis dissimilarity indexes were observed, suggesting infant gut microbiota composition was not more similar to MOM compared to a random milk sample (*p* = 0.301; Figure S4C). Finally, generalized Procrustes analysis was used to integrate all three datasets, showing no significant relationship between any sample types (Figure S4D; HMO profile-MOM microbiota, *p* = 0.076; HMO profile-infant gut microbiota, *p* = 0.645; MOM microbiota-infant gut microbiota, *p* = 0.189).

## Discussion

MOM feeding is protective against NEC, and multiple factors might contribute to this protection. MOM may shape the infant gut microbiome indirectly by providing HMOs and other bioactive components, and/or directly by delivering potential gut colonizers. Here, we investigated the MOM microbiota in NEC and integrated MOM microbiota, MOM bacterial load, HMO profiles, and infant gut microbiota in a cohort of preterm infants. No statistically significant differences were found in MOM microbiota or bacterial load between infants diagnosed with definite NEC compared to healthy controls, either in the entire dataset or in the stratified cohorts. Contrary to what has been reported for term infants, where HMO profiles are linked to the relative abundance of specific taxa,^25^^,^^26^ no correlation between HMOs and preterm infant gut bacteria was found in our exclusively preterm cohort, suggesting other neonatal intensive care unit (NICU) processes dominate changes in the preterm gut microbiota.^27^ There was however a significant positive correlation in the relative abundance of *Acinetobacter* in MOM and infant gut microbiota. Overall, in preterm infants cared for in NICU, our results suggest the MOM microbiota does not impact NEC risk.

Owing to the samples being low biomass, amplicon-based 16S rRNA gene sequencing, as opposed to non-amplicon metagenomics, was applied to study the MOM microbiota. As observed in previous term and preterm studies, *Staphylococcus* was the most abundant genus found in MOM samples.^28^^,^^29^ *Staphylococcus* is a skin colonizer, and translocation of the bacterium from the breast skin to the mammary gland or directly in the MOM upon expression could be expected. While few studies have sequenced the microbiota in MOM from preterm infants, the abundant genera in our UK cohort were generally consistent with a previous large study in a Canadian cohort (*n* = 86 infants), where the top 10 most abundant genera included *Staphylococcus*, *Acinetobacter*, *Pseudomonas*, *Corynebacterium*, *Finegoldia*, and *Streptococcus*.^28^ In contrast, the other most abundant genera found in the current cohort were *Enterococcus*, *Enterobacter*, *Cutibacterium*, and *Bifidobacterium.* Notably, probiotics containing *Bifidobacterium* were used in the current cohort, but not the Canadian study. We have shown that probiotic receipt dominates differences in the preterm gut microbiome^27^ and the cross-colonization of probiotic species can occur across the NICU.^30^

The current study found no differences in MOM microbiota or bacterial load between healthy and NEC infants in an exclusively preterm cohort cared for in NICU. Previous work has shown that the HMOs profiles in MOM received by preterm infants who developed NEC are different from those received by healthy controls; in particular, MOM received by NEC infants has a lack of DSLNT.^6^^,^^9^^,^^10^ Taken together, the lower DSLNT concentration in NEC does not appear to be a consequence of altered MOM microbiota, or vice versa. Given the importance of human milk for preterm infant health, donor human milk feeding in preterm infants is increasingly used in routine clinical practice to make up MOM shortfall. Implementing donor human milk is safe and has generally been associated with a reduced risk of developing NEC.^31^^,^^32^^,^^33^^,^^34^ While pasteurization of human milk removes viable microbes, HMOs are largely unaltered and other bioactive components are variably impacted.^35^^,^^36^ In accordance with MOM microbes having no association with NEC, experimental work with Caco-2 epithelial cells has further showed that host interleukin-8 response to donor human milk is not impacted by restoring the MOM microbiota.^23^ This provides further evidence that human milk bioactive components improve infant health through mechanisms independent of the milk, most likely by modulating infant microbiome and/or direct interaction with the host. This has important implications for the rationale of personalizing donor human milk by “seeding” bacteria from small volumes of MOM to restore viable microbes.^21^^,^^22^^,^^23^ Thus, based on the available evidence, efforts to “restore” the MOM in donor human milk are unlikely to provide additional protection against NEC.

We found no link between any single HMO and specific taxa in MOM microbiota. Published studies in term infants have found potential correlations between specific HMOs and milk microbiota; however, the results vary, and no consistent correlation has been identified.^17^^,^^18^^,^^19^ In contrast to the MOM microbiota, the impact of HMOs on the term infant gut microbial community has been more widely studied, with the most consistent correlation reporting that HMO consumption shapes a gut rich in bifidobacteria when compared to infants receiving formula only.^25^^,^^37^^,^^38^^,^^39^ All preterm infants included in our study received MOM, preventing analysis exploring the potential microbiota shaping effect exerted by receipt of HMOs compared to formula. The lack of correlation between specific HMOs and the *Bifidobacterium* genus in this preterm cohort may reflect infants receiving antibiotics at birth and during the first weeks of life, which are reported to reduce bifidobacteria in the gut,^24^^,^^40^ combined with the near-universal use of probiotics (97%) containing *Bifidobacterium* spp. that may also have masked potential correlation between HMOs and *Bifidobacterium*. The strain-to-strain variability of HMO utilization by different *Bifidobacterium*, which could not be disentangled in this current study, may also mask potential correlations between HMOs and *Bifidobacterium* at lower taxonomic levels.

Vertical transmission of MOM bacteria to the infant gut has been reported in term infants.^13^^,^^41^ In our study, a positive correlation was found between *Acinetobacter* in MOM and infant gut, while a negative correlation was found between infant gut *Acinetobacter* and MOM *Staphylococcus*. This likely reflects MOM high in *Acinetobacter* was low in *Staphylococcus*. That high *Acinetobacter* in MOM correlated with higher *Acinetobacter* in the infant gut, but higher *Staphylococcus* was not correlated with higher *Staphylococcus* in the infant gut, may reflect the antimicrobial resistance harbored by *Acinetobacter* or that *Staphylococcus* is transmitted through other routes (e.g., maternal and NICU staff skin). Notably, *Acinetobacter* has consistently been reported as one of the abundant bacteria in MOM from both preterm^28^^,^^42^ and term cohorts,^43^ and preterm infants are at increased risk of developing sepsis caused by *Acinetobacter* spp.^44^^,^^45^

### Conclusion

Recent work has highlighted the importance of human milk bioactive components and the gut in preterm infants in reducing the risk of NEC. An important missing link was the role of MOM microbiota in directly seeding the infant gut and how this relates to NEC or health. In a large population of preterm infants diagnosed with NEC, and gestational and DOL-matched controls from the same unit, this study finds no evidence that MOM microbiota is associated with the disease. The study further shows only limited correlation between bacterial genera in MOM and infant gut, and no correlation between any bacterial genera and specific HMOs. Taken together, our data suggest MOM is protective against NEC through mechanisms other than the MOM microbiota, such as provision of bioactive components that can either act directly on the host or act indirectly through modulation of the microbiota. Thus, efforts to “restore” MOM microbes in donor human milk may not provide additional protection against NEC. The focus should instead be on increasing fresh MOM intake and discovering NEC therapies independent of MOM microbiota, such as human milk bioactive components.

### Limitations of the study

Limitations that should be considered include the following. First, this study used one sample per mother-infant pair, preventing analysis of temporal dynamics. Owing to the lack of agreed definitions of suggested NEC subtypes and that large case numbers would be required to enable such stratification, NEC was analyzed as a single outcome but likely represents the common endpoint of different etiologies. MOM samples may have been subject to a freeze/thaw cycle before being fed to the infant and were collected from the feeding tubes at the end of feeds, which may impact microbial viability. Nonetheless, the samples reflect exactly what the infant was exposed to, and our methods were primarily analyzing both live and dead cells. Despite our efforts, we were unable to find other researchers/cohorts with a meaningful number of MOM samples from infants who developed NEC, nor any published or publicly available data. Moreover, almost all infants were supplemented with probiotics containing *Bifidobacterium* spp. and all received some MOM, potentially masking correlations between diet-microbe interaction in the cohort. While the volume of MOM may impact findings, this is highly variable on a day-to-day basis and difficult to include in a meaningful way in analysis. Detailed maternal information was not available for variables that may impact both HMOs and MOM microbiota (diet, body mass index, etc.), but this would not impact the overall conclusions. Finally, strain characterization for the MOM and infant stool was not possible and would be required to prove direct transmission of the bacteria from the mother to the infant, which may reveal other MOM microbiota interactions.

## Resource availability

### Lead contact

Further information and requests for resources and reagents should be directed to and will be fulfilled by the lead contact, Christopher Stewart (christopher.stewart@newcastle.ac.uk).

### Materials availability

This study did not generate new unique reagents.

### Data and code availability

All sequencing data generated and analyzed in this study have been deposited in the European Nucleotide Archive. The MOM microbiota data and corresponding metadata are available under study accession number PRJEB72536. The infant gut microbiome data and corresponding metadata are available under study accession number PRJEB39610. This study used pre-existing software and did not generate new custom code. Any additional information required to reanalyse the data reported in this paper is available from the lead contact upon request.

## Acknowledgments

The authors wish to thank the neonatal intensive care staff involved in the sample collection, particularly Julie Groombridge. We are grateful to the families for their willingness to help and support research. We also thank Daniel Smith, Kristi Hoffman, Matt Wong, and Joseph Petrosino (Baylor College of Medicine) for support with bioinformatic progressing of raw data.

This work was supported by the Sir Henry Dale Fellowship jointly funded by the 10.13039/100010269Wellcome Trust and the 10.13039/501100000288Royal Society (grant number 221745/Z/20/Z), a Newcastle University Academic Career Track (NUAcT) Fellowship, and the 2021 Lister Institute Prize Fellow Award, awarded to C.J.S. For the purpose of open access, the authors have applied a CC BY public copyright license to any author-accepted manuscript version arising from this submission.

The funders played no part in the study design, analysis, interpretation, or reporting.

Correspondence should be addressed to Janet Berrington and Christopher Stewart. All sequencing data generated and analyzed in this study have been deposited in the European Nucleotide Archive. The MOM microbiota data are available under study accession number PRJEB72536. The infant gut microbiome data are available under study accession number PRJEB39610.

## Author contributions

N.D.E., J.E.B., and C.J.S. conceived and designed the study. J.E.B. oversaw the sample collection, and G.R.Y. and C.J.S. oversaw storage logistics. A.C.M., A.H., J.D.P., and L.B. produced the data. A.C.M. and L.C.B. performed the analysis. N.D.E., J.E.B., and C.J.S. supervised the study. A.C.M., J.E.B., and C.J.S. cowrote the manuscript, and all authors approved the final submission.

## Declaration of interests

L.B. is the UC San Diego Chair of Collaborative Human Milk Research, endowed by the Family Larsson-Rosenquist Foundation (FLRF), Switzerland. L.B. is a co-inventor on patent applications related to the use of HMOs in preventing NEC and other inflammatory diseases. N.D.E. and J.E.B. report grants to their institutions from Prolacta Bioscience US and Danone Early Life Nutrition. N.D.E. declares lecture honoraria donated to charity from Nestlé Nutrition Institute. C.J.S. declares lecture honoraria from Nestlé Nutrition Institute.

## STAR★Methods

### Key resources table

**Table:** 

REAGENT or RESOURCE	SOURCE	IDENTIFIER
**Critical commercial assays**		

DNeasy PowerLyzer PowerSoil Kit	QIAGEN	Cat. No. 12855
PowerUp™ SYBR™ Green Master Mix	Applied Biosystems	Cat. No. A25777
deMan Rogosa Sharpe	BD Difco	Cat. No. BD 288130
Brain Heart Infusion	Millipore	Cat. No. 53286
CHROMID® CPS® Elite	bioMerieux	Cat. No. 418284
Fastidious Anaerobe Agar	Thermo Scientific	Cat. No. PB0225A
MacConkey Agar	Millipore	Cat. No. M7408
MacConkey Agar n. 3	Thermo Scientific	Cat. No. PO0495A

**Deposited data**		

Raw 16S rRNA gene sequencing data for MOM microbiome	This paper	ENA: PRJEB72536
Raw metagenomic data from infant gut microbiome	(Masi et al.)^10^	ENA: PRJEB39610

**Oligonucleotides**		

qPCR primers for total bacteria load	(Liu et al.)^46^	N/A

**Software and algorithms**		

BBMap version 38.82	(Truong et al.)^47^	https://sourceforge.net/projects/bbmap/
vsearch	(Rognes et al.)^48^	https://github.com/torognes/vsearch
UPARSE	(Edgar et al.)^49^	https://drive5.com/uparse/
USEARCH	(Edgar et al.)^50^	https://drive5.com/usearch/
UCHIME	(Edgar et al.)^50^	https://drive5.com/usearch/manual/uchime_algo.html
psych version 2.2.5	(Ravelle)^51^	https://cran.r-project.org/web/packages/psych/index.html
vegan version 2.6–4	Oksanen et al.^52^	https://cran.r-project.org/web/packages/vegan/index.html
R environment version 3.6.3	R core Team	https://www.r-project.org/

### Experimental model and study participant details

#### Ethics and samples collection

Preterm infants (born at <32 weeks gestation) were born or transferred to a single tertiary level Neonatal Intensive Care Unit in Newcastle upon Tyne, UK, and participated in the Supporting Enhanced Research in Vulnerable Infants (SERVIS) study (REC10/H0908/39) after written informed parental consent. Diagnoses were made using an extensive combination of clinical, X-ray and histological findings and blindly agreed by two neonatal clinicians. Necrosis of the bowel was confirmed through histology for infants who underwent surgery.

#### Population description and clinical data and code availability

Demographic data and clinical diagnoses were made in a standardised way as previously described.^53^ Standard clinical protocols recommended the routine use of supplemental probiotics when minimal enteral feeds were tolerated. Feeds were started with MOM and increased with 24 mL/kg/day once initial milk was tolerated, and total parenteral feed was stopped once milk feeds of 150 mL/kg/day were tolerated enterally. Further information on clinical practice in the NICU can be found in the study published by Granger et al..^53^ The probiotics administered were Labinic (*Lactobacillus acidophilus*, *Bifidobacterium infantis* and *B. bifidum*) or Infloran (*L. acidophilus* and *B. bifidum*). Control infants were healthy (defined as no NEC or focal perforation), matched by gestation, and chosen by availability of relevant samples.

### Method details

#### Breast milk samples collection

Breast milk samples were collected from residual from infant’s feeding systems. After collection for research, samples were stored in sterile tubes. All samples were stored in the NICU at −20°C before being transferred and stored at −80°C. Infants may receive fresh breast milk on the day of expression, or frozen breast milk. The day of MOM sample reflects the day the infant received the milk. Stool was collected from the nappy/diaper and stored at −20°C before transfer to −80°C.

#### DNA extraction and 16S rRNA gene sequencing of mother’s own milk

DNA was extracted for downstream analysis using the DNeasy PowerLyzer PowerSoil Kit (QIAGEN) following the manufacturer’s instructions with some modifications to maximise yield given the relative low sample volume and low biomass nature of MOM. 400 μL of MOM sample were added to the PowerBead tube and mixed with 400 μL of PowerBead solution and 60 μL of solution C1. Where 400 μL of samples were not available, a sample volume as low as 150 μL was used. Samples were vortexed at maximum speed for 20 min using a Vortex Adapter tube holder. The tubes were subsequently centrifuged at 10,000 g for 1 min, the supernatant moved to a clean tube. Solutions C2 and C3 were mixed in 1:1 proportion, and 200 μL of the mix were added before a unique 5 min incubation at 4°C. The subsequent steps were performed as per the protocol instructions. Finally, 60 μL of Solution C6 was added to the column for 5 min at room temperature and then centrifuged at 10,000 g for 1 min and the samples stored at −80°C. A negative control was extracted in every batch of 24 samples.

Polymerase chain reaction (PCR) was used to amplify the V4 region of the 16S rRNA gene using the barcoded Illumina adapter-containing primers 515F and 806R.^54^ Sequencing was performed on the MiSeq platform (Illumina), with a target read depth of 10k and a paired end read length of 250 bp. Raw fastq files were demultiplexed using the Illumina ‘blc2fastq’ software, followed by quality trimming and Illumina adapters and PhiX reads removal using bbduk (BBMap version 38.82).^47^ Reads with a Phred quality score below 15 and length below 100 bp after trimming were discarded. Reads are then merged using bbmerge (BBMap version 38.82),^47^ with subsequent further filtering using ‘vsearch’^48^ setting the maximum expected error of 0.05, maximum length of 254 bp and minimum length of 252 bp. Using the UPARSE algorithm,^49^ sequences were clustered into Operational Taxonomic Units (OTUs) applying a similarity cut-off of 97% and using a stepwise approach. USEARCH and UCHIME^50^ were then used to remove chimeras, and USEARCH^55^ was then used to determine taxonomies by mapping the OTUs against the SILVA Database version 138.1^56^ containing only the 16S V4 region. Abundances were then recovered by mapping the demultiplexed reads to the OTUs file and were then used for subsequent analysis.

#### Quantitative polymerase chain reaction to determine total bacterial load

Quantitative polymerase chain reaction (qPCR) was used to determine the total bacterial load of MOM sample using the primers developed by Liu et al. (2012) targeting a 466 bp fragment in the V3-V4 region of the 16S rRNA gene.^46^ 20μL reaction volume was used, composed of 10μL of PowerUp SYBR Green Master Mix (Applied Biosystems), a final primer concentration of 1.8μM (forward: 5′- CCTACGGGDGGCWGCA-3′, reverse: 5′- GGACTACHVGGGT MTCTAATC -3′) and 1μL of template DNA. A standard curve was created by amplifying the region from an *Escherichia coli* strain and creating 10-fold dilutions ranging 10^9^-10^2^ copies/μL. A negative control was included in each reaction. The reaction was performed using the StepOne Plus Real-Time machine (Thermo Fisher) with the following conditions: 10 min at 95°C, followed by 30 cycles of 15 s at 95°C and 1 min at 60°C.

#### Bacterial isolation

Samples high in specific genera according to 16S rRNA gene sequencing were selected for culturing in order to determine the viability of the bacteria in this sample type. Serial dilutions of MOM samples in PBS were prepared and 100 μL of 10^−1^, 10^−3^ and 10^−5^ dilutions were cultured on several culture media. Media used for isolation included deMan, Rogosa, Sharpe (MRS) supplemented with 50 mg/mL of L-cysteine HCl and with or without 50 mg/mL mupirocin; Bifidus Selective Media (BSM); TOS-propionate agar medium; Brain Heart Infusion (BHI); Fastidious Anaerobe Agar (FAA); CHROMID CPS Elite; MacConkey Agar, MacConkey Agar n.3. After plating, the samples were left incubating at 37°C for up to 96h. Colonies with diverse morphology were sub-cultured at least twice to obtain pure single colonies, which were then grown in liquid media. In case of isolation of anaerobic bacteria, every step was performed in anaerobic atmosphere [∼60ppm O_2_, 2.5% H_2_] at 37°C in a Coy Type B Anaerobic Chamber and agar plates, broth media and liquid reagents were left in the anaerobic chamber overnight before usage. In case of isolation of aerobic bacteria a tissue culture incubator at 37°C and 5% CO_2_ was used. rRNA gene sequencing (27F 5′-AGAGTTTGATCCTGGCTCAG3’; 1492R 5′-GGTTACCTTGTTACGACTT-3′) and/or by matrix-assisted laser desorption ionization-time of flight mass spectrometry (MALDI-TOF MS) of single fresh colonies were used to identify isolates.

### Quantification and statistical analysis

#### Statistical analysis of mother’s own milk 16S rRNA gene sequencing data

A total of 3,898,316 mapped reads were obtained from the 16S rRNA gene sequencing of 130 MOM DNA samples. Each sample was rarefied to 1231 reads and a total of 111 samples were included in the final analysis (median 22,560 reads per sample). The data analysis was performed using the Agile Toolkit for Incisive Microbial Analysis (ATIMA; https://atima.research.bcm.edu/). In accordance with the stool metagenomic data, alpha diversity analysis was performed based on observed species (richness) and Shannon diversity (richness and evenness). Beta-diversity was performed using Bray-Curtis principal coordinate analysis, with statistical significance between groups determined using PERMANOVA. Mann-Whitney test (two group comparison) and Kruskal-Wallis test (three group comparison) were performed to assess differential abundance at phylum and genus level, and *p*-values were adjusted using the false discovery rate (FDR) algorithm.

#### Integration of human milk oligosaccharide profile data, stool metagenomes, and mother’s own milk 16S rRNA gene sequencing data

Spearman correlation analysis was performed between the 3 possible pairs of comparison (HMO profile and MOM microbiota, HMO profile and infant microbiota, and MOM microbiota and gut microbiota). The analysis was performed in R (version 3.6.3) using the “psych” package (version 2.2.5),^51^ and *p* values were adjusted using the FDR algorithm.

Generalised procrustes analysis was used to integrate all three datasets using the vegan package (version 2.6–4)^52^ and *p* values were determined using the “protest” function. Bray-Curtis dissimilarity indexes between MOM microbiota and infant gut microbiota were determined using the vegan package (version 2.6–4).^52^ Wilcoxon test was applied to compare dissimilarity indexes between matched mother-infant samples and one random unmatched mother-infant sample per each pair.

## Supplemental information

Document S1. Figures S1–S4 and Table S1

Document S2. Article plus supplemental information
