# Supplementary material for: Human milk microbiota, oligosaccharide profiles, and infant gut microbiome in preterm infants diagnosed with necrotizing enterocolitis
Source: Cell Rep Med. 2024 Aug 30;5(9):101708. doi: 10.1016/j.xcrm.2024.101708 (PMC11524953; doi:10.1016/j.xcrm.2024.101708)
Supplement: Document S1. Figures S1–S4 and Table S1 [file mmc1.pdf]

Cell Reports Medicine, Volume *5*

Supplemental information

Human milk microbiota, oligosaccharide profiles, and infant gut microbiome in preterm infants diagnosed with necrotizing enterocolitis

Andrea C. Masi, Lauren C. Beck, John D. Perry, Claire L. Granger, Alice Hiorns, Gregory R. Young, Lars Bode, Nicholas D. Embleton, Janet E. Berrington, and Christopher J. Stewart

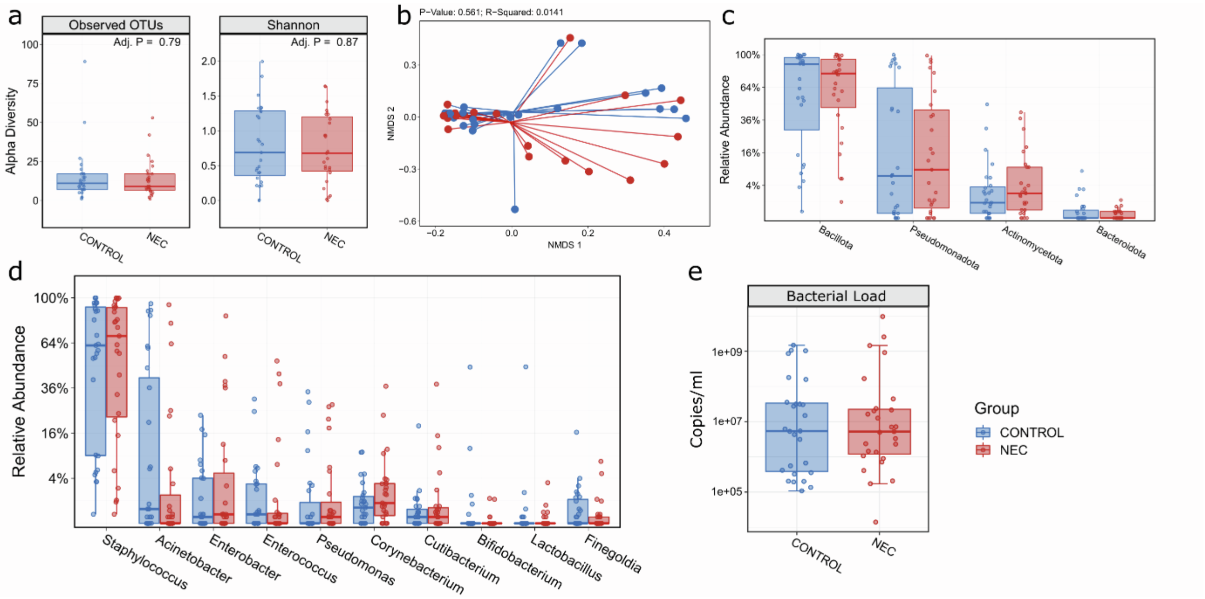

Supplementary Figure 1. Analysis of mother's own milk microbiota from samples collected before NEC diagnosis and matched controls. (a) Box plots showing the alpha diversity based on observed OTUs (richness) and Shannon diversity. P values were calculated by applying the Mann-Whitney test and adjusted using the FDR algorithm. (b) NMDS plot of weighted Bray-Curtis dissimilarity. P value based on PERMANOVA. Box plots showing the relative abundance of phyla (c) and the top 10 most abundant genera (d). (e) Box plot of the total bacterial load. P values were calculated by applying the Mann-Whitney test and adjusted using the FDR algorithm. Adjusted P values in panels c and d were all >0.05. A total of 27 NEC and 27 matched controls were included. Related to Figure 2.

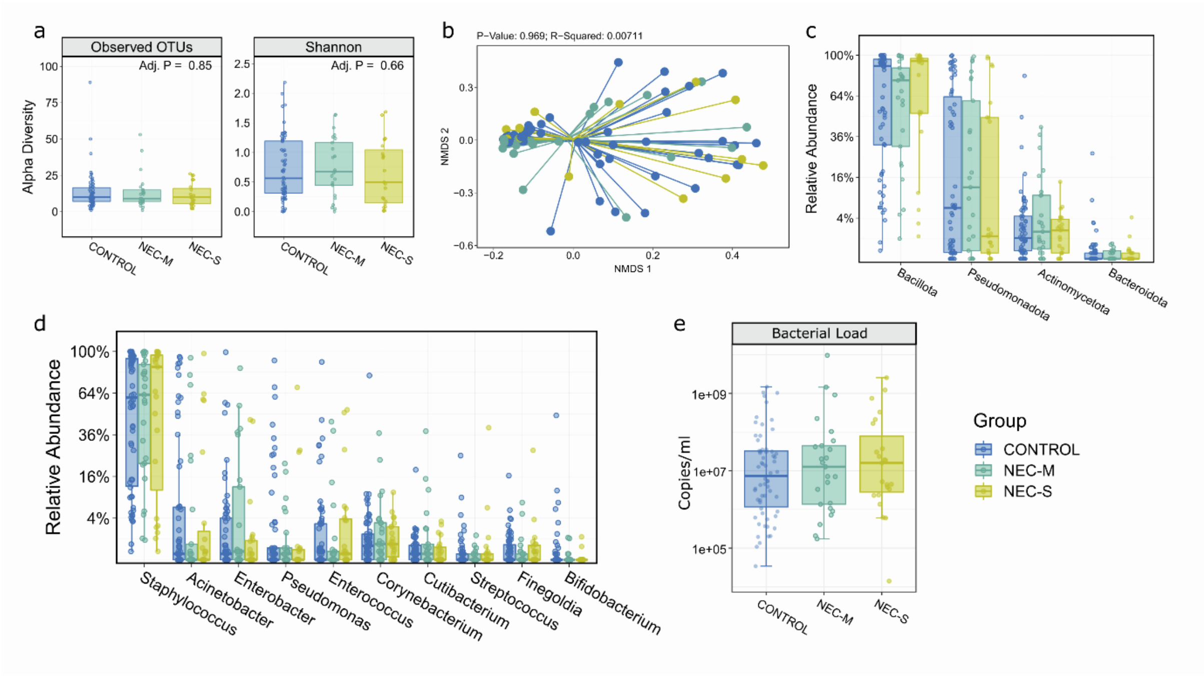

Supplementary Figure 2. Cross-sectional analysis of preterm MOM 16S rRNA gene profile with stratification of surgically managed NEC (NEC-S) and medically managed NEC (NEC-M). (a) Box plots showing the alpha diversity based on observed OTUs (richness) and Shannon diversity. P values were calculated by applying the Kruskal-Wallis test and adjusted using the FDR algorithm. (b) NMDS plot of weighted Bray-Curtis dissimilarity. P value based on PERMANOVA. Box plots showing the relative abundance of phyla (c) and the top 10 most abundant genera (d). (e) Box plot of the total bacterial load. All P values were calculated by applying the Kruskal-Wallis test and adjusted using the FDR algorithm. Adjusted P values in panels c and d were all >0.05. A total of 48 NEC and 62 control infants were included. Related to Figure 2.

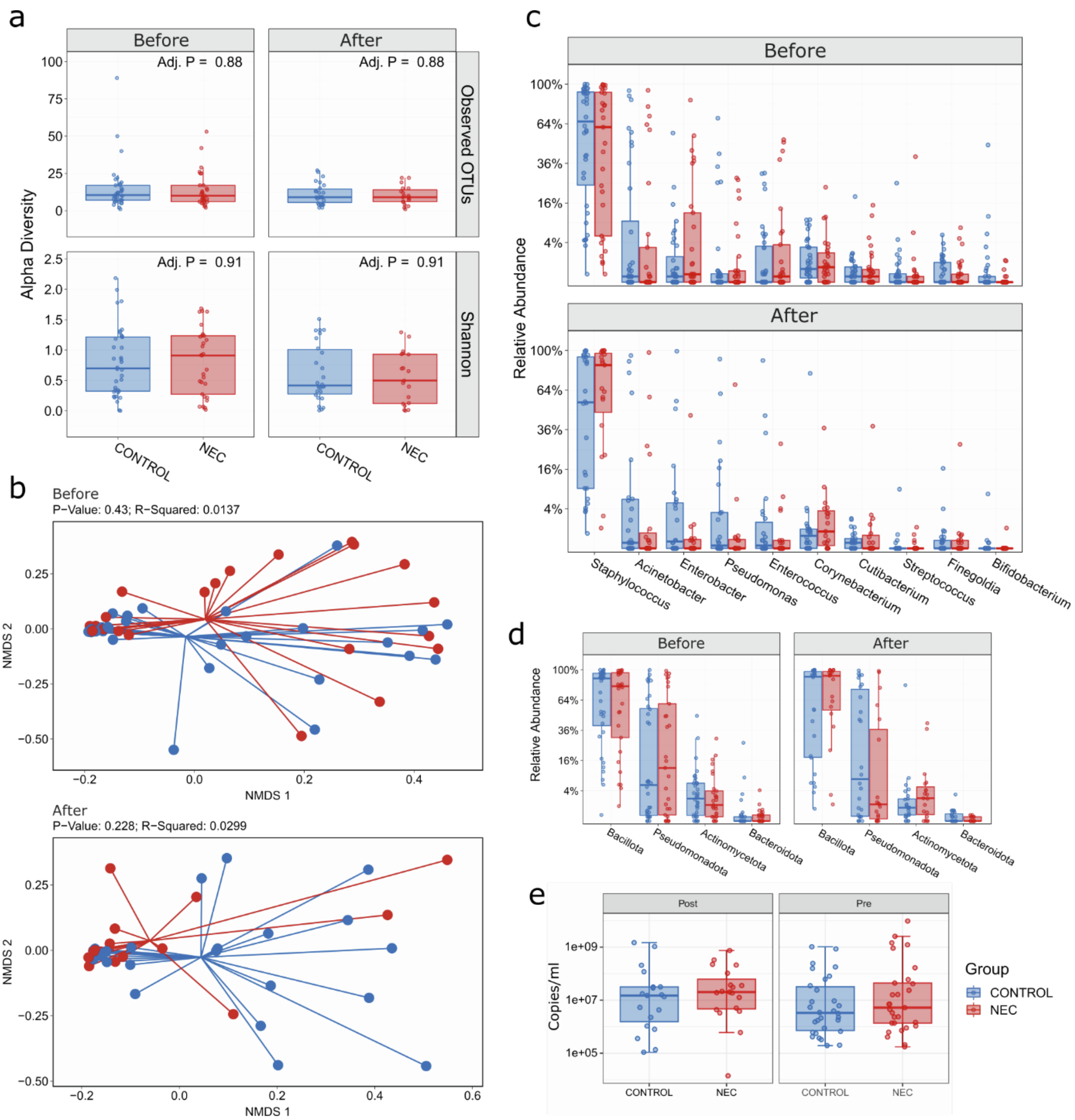

Supplementary Figure 3. Cross-sectional analysis of preterm MOM microbiota with stratification in relation to achieving full enteral feeds**.** (a) Box plots showing the alpha diversity based on observed OTUs (richness) and Shannon diversity. P values were calculated by applying the Mann-Whitney test and adjusted using the FDR algorithm. (b) NMDS plot of weighted Bray-Curtis dissimilarity. P value based on PERMANOVA. Box plots showing the relative abundance of phyla (c) and the top 10 most abundant genera (d). (e) Box plot of the total bacterial load. P values were calculated by applying the Mann-Whitney test and adjusted using the FDR algorithm. Adjusted P values in panels c and d were all >0.05. A total of 29 NEC and 29 matched control infants were included in the pre-full feed analysis; 19 NEC and 19 matched control infants were included in the post-full feed analysis. Related to Figure 2.

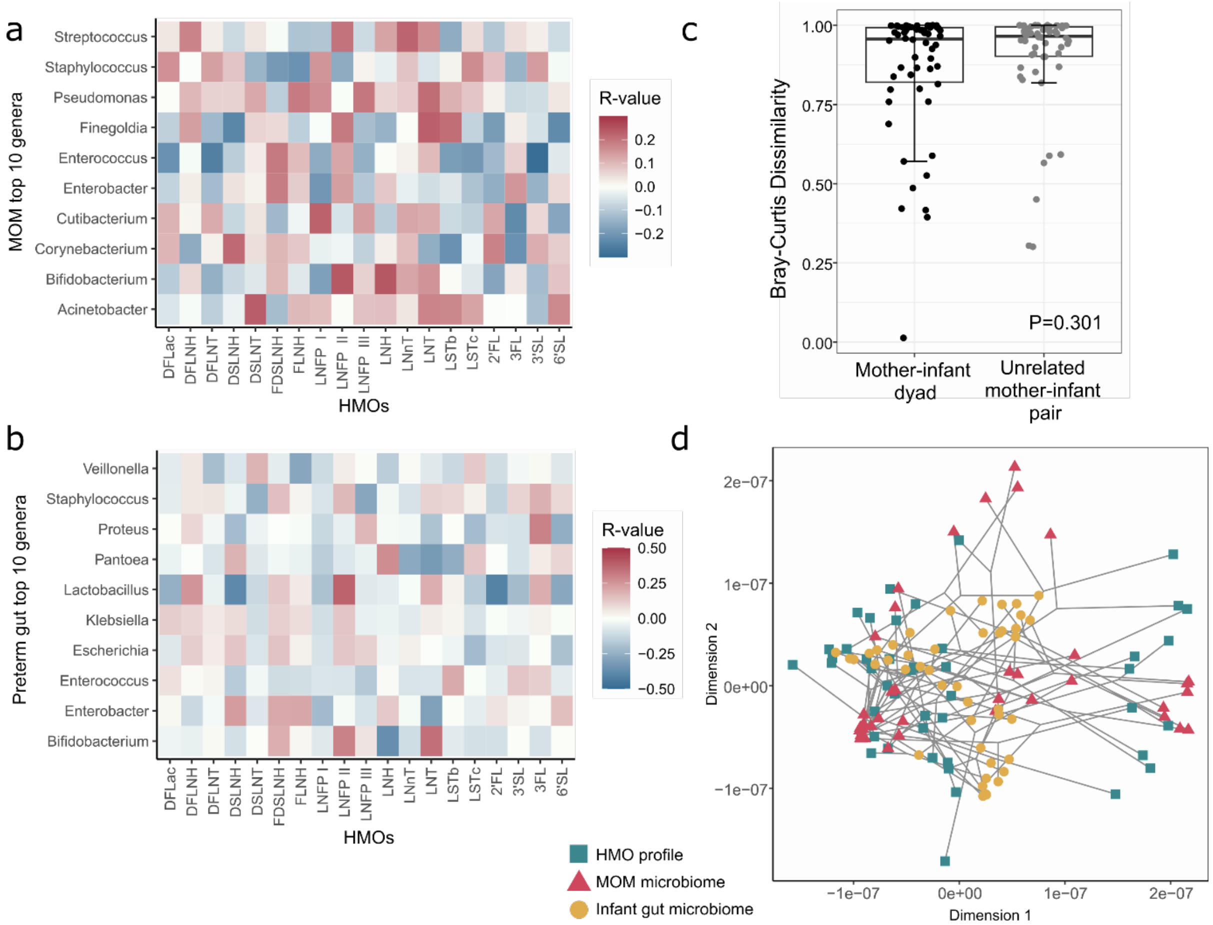

Supplementary Figure 4. Integrated analysis of HMO profiles, MOM microbiota and infant gut microbiota. Heatmap showing the R2 value for Spearman correlation analysis between HMO concentration and the top 10 most abundant genera in MOM (65 preterm infants were included) (a) and top 10 most abundant genera in preterm gut microbiota (51 preterm infants included) (b). R2 values and P values were calculated using the "psych" package in the R environment and P values were adjusted using the FDR algorithm. All adjusted P value were >0.05. (c) Boxplot showing the Bray-Curtis dissimilarity indexes calculated between mother-infant dyads compared to a randomly paired, unrelated, mother and infant samples. P value was calculated using Wilcoxon test. (d) Procrustes analysis based on superimposition of the PCoA coordinates of HMO profile (square), MOM microbiota (triangle) and infant gut microbiota (circle) (45 preterm infants were included). Matched samples from each mother-infant dyad are connected by lines, where shorter lines represent greater the correlation between the datasets. Related to Figure 3.

Supplementary Table 1. Microbial isolates cultured from mother's own milk. Related to "Bacterial isolation" paragraph in STAR Methods section.

**Species**	**N samples positive in culturing**	**Agar media the species could be isolated from**
Staphylococcus epidermidis	11	CPSE, BHI, BHI + 5% blood, TOS, MRS-, FAA, BSM, MacConkey, MacConkey n.3
Cutibacterium acnes	8	FAA, BSM, MRS+, MRS-
Enterococcus faecalis	6	CPSE, BHI, BSM, TOS, MRS-, FAA, MRS+, MacConkey
Bifidobacterium breve	3	BSM, MRS-, MRS+, TOS
Enterobacter hormaechei	3	BSM, BHI + 5% blood, MacConkey, MacConkey n.3 BHI, CPSE
Staphylococcus lugdunensis	3	BHI, BHI + 5% blood, TOS, MacConkey, FAA
Stenotrophomonas maltophilia	3	YPD + P/S, MacConkey, MacConkey n.3, CHROMagar Acinetobacter
Acinetobacter ursingii	2	MacConkey, MacConkey n.3
Bifidobacterium animalis	2	BSM, TOS, MRS+, MRS-
Cutibacterium avidum	2	BSM
Delftia lacustris	2	MacConkey n.3, CHROMagar Acinetobacter
Enterobacter roggenkampii	2	BSM, BHI + 5% blood, MacConkey, MacConkey n.3 BHI, CPSE
Pantoea septica	2	MacConkey
Staphylococcus aureus	2	CPSE, BSM
Staphylococcus hominis	2	CPSE, BHI, MacConkey, BHI + 5% blood
Acinetobacter beijerinckii	1	MacConkey
Acinetobacter pittii	1	MacConkey
Anaerococcus senegalensis	1	FAA
Atlantibacter hermannii	1	YPD + P/S
Bifidobacterium bifidum	1	MRS-
Bifidobacterium longum	1	MRS+
Candida parapsilosis	1	BHI, BHI + 5% blood, CPSE
Corynebacterium kroppenstedtii	1	BHI

**Species**	**N samples positive in culturing**	**Agar media the species could be isolated from**
Corynebacterium pyruviciproducens	1	BHI + 5% blood
Delftia acidovorans	1	MacConkey
Enterobacter bugandensis	1	CPSE
Escherichia coli	1	CPSE
Escherichia hermannii	1	MacConkey n.3
Finegoldia magna	1	FAA
Klebsiella grimontii	1	YPD + P/S
Lactobacillus gasseri	1	MRS-
Pseudomonas fulva	1	CHROMagar Acinetobacter
Pseudomonas lactis	1	YPD + P/S
Pseudomonas oryzihabitans	1	MacConkey n.3
Pseudomonas plecoglossicida	1	YPD + P/S
Schaalia radingae	1	FAA
Sphingomonas paucimobilis	1	YPD + P/S
Staphylococcus capitis	1	BHI
Staphylococcus condimenti	1	CPSE
Staphylococcus haemolyticus	1	CPSE
Staphylococcus warneri	1	BSM, BHI
Stenotrophomonas rhizophila	1	YPD + P/S
Streptococcus anginosis	1	BHI

BHI, brain heart infusion; MRS, De Man, Rogosa and Sharpe; MRS+, MRS supplemented with L-cysteine and mupirocin; MRS-, MRS supplemented with L-cysteine; TOS, transgalctosylated oligosaccharide; BSM, bifidus Selective Medium; FAA, fastidious anaerobe agar; YPD, yeast extract peptone dextrose; P/S, penicillin and streptomycin supplement.
